# Population-based outcomes after brain radiotherapy in patients with brain metastases from breast cancer in the Pre-Trastuzumab and Trastuzumab eras

**DOI:** 10.1186/1748-717X-8-12

**Published:** 2013-01-09

**Authors:** Irene Karam, Sarah Hamilton, Alan Nichol, Ryan Woods, Caroline Speers, Hagen Kennecke, Scott Tyldesley

**Affiliations:** 1 University of British Columbia, Vancouver, BC, Canada; 2 Radiation Therapy Program, BC Cancer Agency – Vancouver Centre, 600 west 10th Avenue, Vancouver, BC, V5Z 4E6, Canada; 3 Cancer Surveillance and Outcomes, Population Oncology, BC Cancer Agency, Vancouver, BC, Canada; 4 Breast Cancer Outcomes Unit, BC Cancer Agency, Vancouver, BC, Canada; 5 Systemic Therapy Program, BC Cancer Agency, Vancouver, BC, Canada

**Keywords:** Breast cancer, Brain metastasis, Brain irradiation, Trastuzumab, HER2 status

## Abstract

**Purpose:**

To evaluate the survival of patients with human epidermal growth factor receptor 2 (HER2) positive and negative metastatic breast cancer irradiated for brain metastases before and after the availability of trastuzumab (T).

**Materials and methods:**

Women diagnosed with brain metastasis from breast cancer in two eras between 2000 and 2007 (T-era, n = 441) and 1986 to 1992 (PreT-era, n = 307), treated with whole brain radiotherapy (RT) were identified. In the T-era, HER2 testing was part of routine clinical practice, and in the preT-era 128/307 (42%) cases had HER2 testing performed retrospectively on tissue microarrays. Overall survival (OS) was estimated using the Kaplan-Meier method and comparisons between eras used log-rank tests.

**Results:**

In the preT- and T-era cohorts, the rate of HER2 positivity was 40% (176/441) and 26% (33/128) (p < 0.001). The median time from diagnosis to brain RT was longer in the preT-era (3.3 years versus 2.3 years, p < 0.001). Survival after brain RT was improved in the T-era compared to the preT-era (1-year OS 26% versus 12%, p < 0.001). The 1-year OS rate for HER2 negative patients was 20% in both eras (p = 0.97). Among HER2 positive patients, the 1-year OS in the preT-era was 5% compared to 40% in the T-era (p < 0.001).

**Conclusions:**

Distinct from patients with HER2 negative disease in whom no difference in survival after brain RT was observed over time, patients with HER2 positive brain metastases experienced significantly improved survival subsequent to the availability of trastuzumab.

## Introduction

The incidence of brain metastases appears to be increasing as women with advanced breast cancer live longer [1]. This is, in part, due to advances in the efficacy of systemic therapy, resulting in improved control of systemic disease, and improved imaging techniques resulting in earlier diagnosis [2]. Historically, median survival (MS) in untreated patients with metastatic disease to the brain has been reported to be one month [3]. Following treatment with whole brain radiotherapy (RT), a median survival of approximately 4–6 months has been reported [3,4].

Human epidermal growth factor receptor 2 (HER2) over-expression has been reported in 20%-25% of human breast cancers and is associated with reduced overall and disease-free survival [1,5]. Studies have demonstrated improved overall survival (OS) and progression-free survival with the use of trastuzumab in combination with chemotherapy in the setting of metastatic breast cancer in 1998 and in the adjuvant setting in 2004 [1,6,7]. Before the availability of trastuzumab, the major factor limiting survival was the progression of systemic disease. Since the introduction of trastuzumab, the proportion of patients with controlled systemic disease dying from cerebral progression has increased [8]. Therefore, it is hypothesized that survival may be improved after whole brain RT in patients with brain metastases from breast cancer in the trastuzumab era and this improvement may be related to improved survival in HER2 positive patients. Further, it is likely that the increase survival maybe due to the impact of trastuzumab, and other diagnostic and therapeutic factors, rather than the biology of HER2 positive disease itself.

This is a report of the clinical characteristics, prognostic variables, and outcomes of patients with HER2 positive and negative metastatic breast cancer who were treated with brain RT before (preT-era) and after (T-era) the availability of trastuzumab.

## Materials and methods

### Study population

The BC Cancer Agency (BCCA) provides all radiation therapy in the province of British Columbia (BC) for a population of approximately 4.5 million. The BC Cancer Registry contains demographic data on all incident cancers, and captures date and cause of death information through a direct linkage to the BC Vital Statistics Agency. In BC, stereotactic radiosurgery (SRS) for patients with brain metastases was available after 1998. Craniotomy has been practiced for decades in the province in selected cases, but it became more widely practiced during the 1990s after publication of the randomized trial by Patchell et al. [9]. HER2 status has been routinely tested in patients with breast cancer since 1999. Trastuzumab became available for patients with metastatic breast cancer outside of a clinical trial setting in August 1998.

A tissue microarray (TMA) of 4,444 patients with a new diagnosis of invasive breast cancer in BC was created from tumour specimens submitted to a central estrogen receptor (ER) laboratory, as previously described [10]. Patients in the TMA cohort were all referred to the BCCA and represented approximately 60% of all breast cancer patients diagnosed in the province during 1986–1992. Single archival blocks were retained at the Vancouver Hospital ER laboratory from each specimen received for ER testing. For both the TMA subjects and patients with clinical HER2 testing since 1999, HER2 status was determined using an immunohistochemistry (IHC) technique. All IHC 2+ and indeterminate cases were tested for gene amplification by fluorescence *insitu* hybridization (FISH). Tumors demonstrating no or 1+ staining by IHC and/or no gene amplification by FISH were scored as HER2 negative, whereas tumors demonstrating 3+ staining by IHC and/or gene amplification by FISH were scored as HER2 positive. When no tumor could be identified on the HER2 slides, the HER2 status was scored as unknown.

A total of 307 women with breast cancer diagnosed between January 1986 and July 1992 (preT-era) and treated with whole brain RT were identified. Excluded from this cohort were patients who had metastatic disease to the skull without brain metastases (n = 2) and whose paper charts were unavailable (n = 2). Of the remaining cases, 128 were in the TMA series and had HER2 testing performed. These 128 patients formed the preT-era cohort for the primary analysis.

Similarly, women were included in the T-era cohort if they had a new diagnosis of breast cancer in BC between January 2000 and December 2007 and they were treated with whole brain RT. Patients who had leptomeningeal disease at the time of whole brain RT (n = 7), metastatic disease to the skull without brain metastases (n = 55) and prophylactic cranial irradiation (n = 5) were excluded. Therefore, a total of 441 patients formed the T-era cohort. Patients diagnosed between 1992 and 2000 could not be included in the analysis as no data on HER2 status was available in that period, as HER2 status analysis was not done as part of routine practice until after 2000.

### Variables

The paper and electronic medical records of all patients were reviewed. Data were collected regarding demographic and clinical characteristics including patient age, M1 stage, grade, hormone receptor status and HER2 status at the initial diagnosis of breast cancer. Brain metastases treatment characteristics included craniotomy use, brain RT dose, chemotherapy, hormone therapy and trastuzumab use on or after the date of first brain metastases. In addition, primary disease control status, existence of extracranial metastases and number of brain lesions at the time of initial brain RT were abstracted. Karnofsky performance status (KPS) (≥70 or <70) was estimated retrospectively at the time point of the initial diagnosis of brain metastases based on narrative notes from attending clinicians in the BCCA chart.

A Recursive Partitioning Analysis (RPA) risk group [11] was determined for each patient prior to initial brain RT. The RPA risk group used four factors: age, KPS, primary controlled/uncontrolled and the presence/absence of extracranial metastases, from which, an RPA class was assigned. Patients with KPS <70 were identified as class 3; patients with KPS ≥70, controlled primary disease, age <65 years and absence of extracranial metastases were identified as class 1 and all other patients were class 2 [11].

To address the potential bias of the TMA cohort from the preT-era not being a subset of the 307 cases in the population-based series, the 128 cases included in the TMA and the 179 cases not included in the TMA cohort were compared for clinical characteristics and survival after brain RT.

### Statistical analysis

All analyses were conducted using the Statistical Package for Social Sciences, version 14.0 (SPSS, Chicago, IL) and the R statistical package, version 2.9.0 (http://cran.r-project.org). Frequencies and descriptive statistics of demographic and clinical variables were obtained. Categorical variables were compared between eras using the Chi-Square test or the Fisher’s Exact test, and continuous variables were compared using the Student *t*-test. Unknown cases were entered into the statistical tests for comparison. Survival from the date of starting brain RT to death or last follow-up was estimated using the Kaplan-Meier method, and survival curves were compared using the log-rank test. Multivariate predictors of survival were determined using the Cox proportional-hazards model for both eras. Variables included in the model were: HER2 overexpression, RPA class, ER status, number of brain metastases, craniotomy, chemotherapy and hormone therapy use, and SRS. Interaction terms were included in the model between era and both hormonal therapy and HER2 as the effects of these two variables were different in the two eras. Age, primary status controlled/uncontrolled, the presence/absence of extracranial metastases, and KPS variables were not included separately in the model, as the RPA class variable is calculated using these variables. Hazard ratios and their 95% confidence intervals (95% CIs) were computed. A *p*-*value* < 0.05 was considered to be statistically significant. This study was approved by the Research Ethics Board of the University of BC.

## Results

### Comparison of outcomes in the preT-era according to inclusion of the patient in the TMA series

In the preT-era, there were 307 women diagnosed with metastatic breast cancer to the brain between January 1986 to July 1992, however, HER2 results are only available for cases included in the TMA for these years. Of the 307 cases, 42% (n = 128) were included in the TMA series and 58% (n = 179) were not included in the TMA series. The age distributions of the TMA and non-TMA subjects, were similar (p = 0.56). Fourteen percent of the non-TMA cases and 4% of the TMA cases had distant metastases at diagnosis. One-year OS was similar in the TMA and non-TMA cohorts (18% and 16%, p = 0.65). All subsequent results reported for the pre-T era refer only to the TMA cohort.

### Patient, tumour and treatment characteristics

Table 1 summarizes patient, tumour and treatment characteristics by era. Median age at diagnosis of brain metastases was 55 years (range, 34–89 years) for the T-era and 53 years (range, 26–77 years) for the preT-era, (p = 0.017). The median time from diagnosis to brain RT was longer in the preT-era (3.3 years versus 2.3 years, p < 0.001). The rates of HER2 positive disease were 40% (n = 176/441) in the T-era and 26% (n = 33/128) in the preT-era cohorts (p < 0.001). In the T-era, among women with HER2 positive disease, 85% (150/176) received trastuzumab after a diagnosis of brain metastases as compared to none in the preT-era. There were no significant differences in other baseline variables (Table 1).

**Table 1:** Comparisons of clinicopathologic and treatment characteristics by era

***Characteristics***	***Pre***-***Trastuzumab Era***	***Trastuzumab Era***	***p-value***
	**n** = **128**	**n** = **441**	
**Age** (**years**)			0.017
<65	104 (81%)	345 (78%)	
≥ 65	24 (19%)	96 (22%)	
Median	53 (26–81)	55 (29–91)	
**M1 at diagnosis**			<0.001
Yes	5 (4%)	113 (26%)	
No	115 (90%)	256 (58%)	
Unknown	8 (6%)	72 (16%)	
**Tumor grade**			0.084
1	0 (0%)	13 (3%)	
2	24 (19%)	106 (24%)	
3	95 (74%)	315 (71%)	
Unknown	9 (7%)	7 (2%)	
**Hormone receptor status**			0.83
ER+	51 (40%)	180 (41%)	
ER-	68 (53%)	249 (57%)	
Unknown	9 (7%)	12 (3%)	
**HER2 status**			<0.001
Positive	33 (26%)	176 (40%)	
Negative	87 (68%)	212 (48%)	
Unknown	8 (6%)	53 (12%)	
**Time from diagnosis to BM** (**years**)			
Median	3.3	2.3	
**KPS at BM**			0.76
<70	47 (37%)	170 (38%)	
≥70	81 (63%)	271 (62%)	
**Number of BM**			0.025
1	44 (34%)	117 (27%)	
2-3	23 (18%)	73 (16%)	
Multiple	61 (48%)	251 (57%)	
**Primary disease at BM**			0.007
Controlled	104 (81%)	305 (70%)	
Uncontrolled	24 (19%)	135 (31%)	
**Extracranial disease at BM**			0.32
Brain only	22 (17%)	97 (22%)	
Bone + brain	22 (17%)	76 (17%)	
Visceral + brain +/−bone	76 (59%)	245 (57%)	
Other	8 (6%)	23 (5%)	
**RPA at BM**			0.17
1	9 (7%)	55 (12%)	
2	72 (56%)	219 (50%)	
3	47 (37%)	167 (39%)	
**Craniotomy**			0.037
Yes	15 (12%)	87 (20%)	
No	113 (88%)	354 (80%)	
Trastuzumab at BM*			
Yes	0 (0%)	150 (34%)	
No	128 (100%)	26 (5%)	
Hormonal therapy at BM*			<0.001
Yes	72 (56%)	100 (23%)	
No	56 (44%)	341 (77%)	
Chemotherapy at BM*			0.032
Yes	43 (34%)	194 (44%)	
No	85 (66%)	242 (55%)	
Unknown	0 (0%)	5 (1%)	

* Refers to treatments given on or after the date of first brain metastases.

*Abbreviations*: *ER* = estrogen receptor, *HER2* = human epidermal growth factor receptor 2, *KPS* = Karnofsky performance status, *BM* = brain metastasis, *RPA* = recursive partitioning analysis.

All women received whole brain RT for their brain metastases. Overall, the three most common prescriptions were 20 Gy in 5 fractions (preT-era: 85% (109/128); T-era: 64% (282/441); p < 0.05), 30 Gy in 10 fractions (preT-era: 4% (5/128); T-era: 24% (105/441); p < 0.05) and 20 Gy in 4 fractions (preT-era: 4% (5/128); T-era: 4% (17/441); p < 0.50). Three percent of women (15/441) had SRS in the T-era as a component of their initial brain metastases treatment.

### Survival

**Figure 1:**
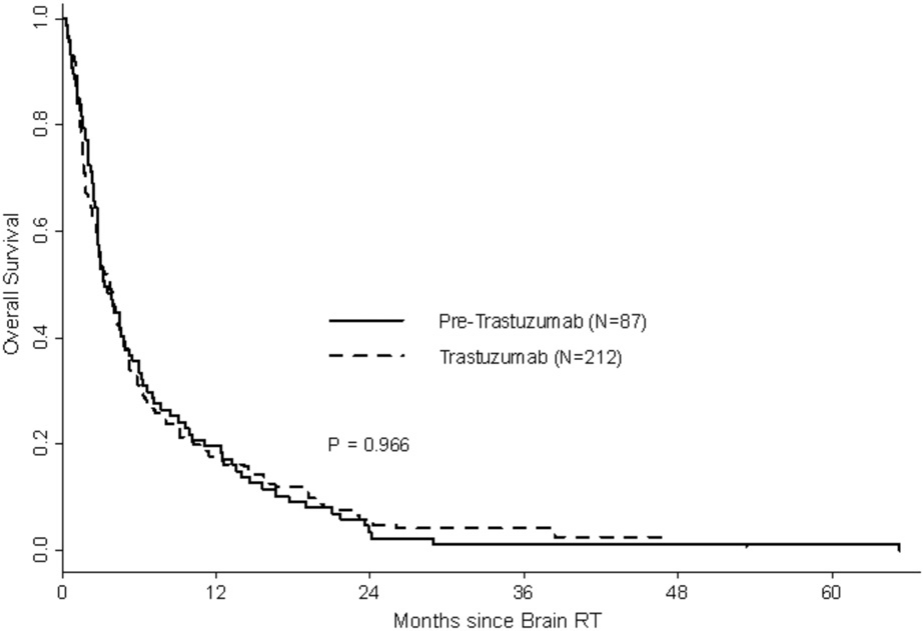
Overall survival by era: HER2 negative patients.

**Figure 2:**
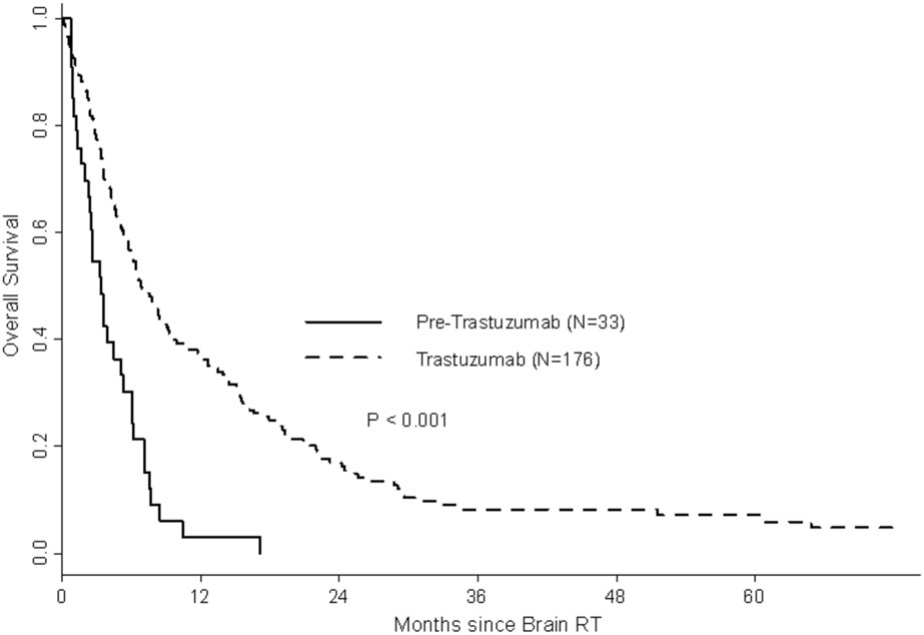
Overall survival by era: HER2 positive patients.

**Figure 3:**
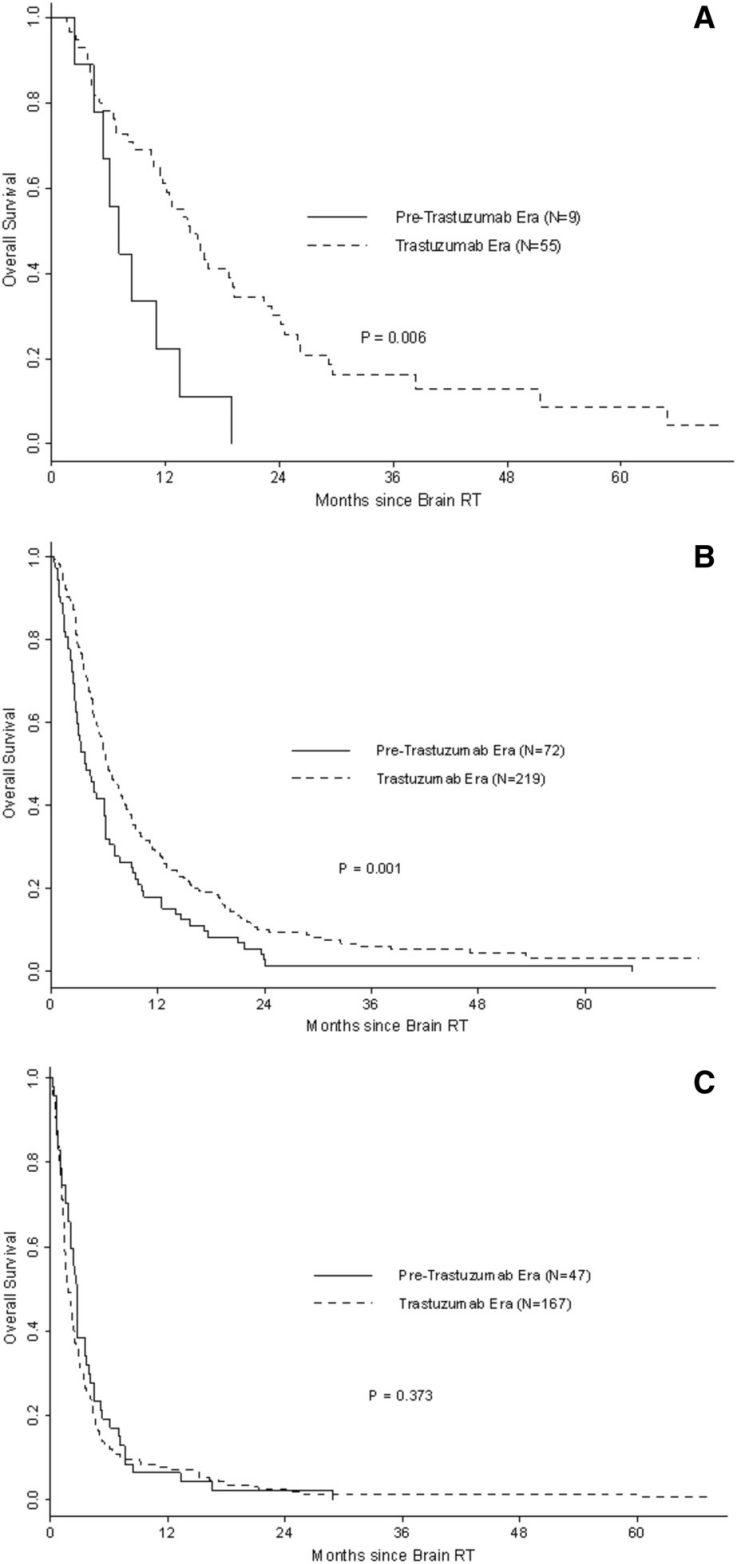
Overall survival by era and RPA class: (A) RPA 1; (B) RPA 2; (C) RPA 3.

One-year OS rates were 12% in the preT-era and 26% in the T-era (p < 0.001). Among patients with HER2 negative disease, 1-year OS rates were 20% for both preT- and T-eras (MS: preT-era: 3.2 months, T-era: 3.7 months, p = 0.97) (Figure 1). Among patients with HER2 positive disease in the preT-era and T-era, 1-year OS rates were 5% and 40% (MS: preT-era: 3.4 months, T-era: 6.8 months, p < 0.001) (Figure 2). Women with RPA class 3 had a median survival in both eras of 1.8 months (preT-era) versus 2.7 months (T-era) (p = 0.37), whereas those with more favourable prognostic features had a longer median survival in the T-era (class 1: preT-era: 6.2 months, T-era: 14.5 months, p = 0.006 and class 2: preT-era: 3.9 months, T-era: 6.4 months, p = 0.001) (Figure 3).

On univariate analysis of patients in the preT-era, RPA class at diagnosis of brain metastases (p = 0.025), use of craniotomy (p < 0.005), the presence of a solitary brain lesion (p < 0.034), and ER positive status (p = 0.013), were associated with improved survival. In the T-era cohort, RPA class at diagnosis of brain metastases (p < 0.001), SRS boost at initial brain metastases (p < 0.001), use of craniotomy (p < 0.001), the presence of a solitary brain lesion (p < 0.001), HER2 overexpression (p < 0.001), chemotherapy use (p < 0.001), hormonal therapy use (p < 0.001) and trastuzumab therapy use (p < 0.001) at brain metastases were favourable prognostic factors for survival.

Table 2 summarizes the results from a multivariable model for OS combining data from both eras. In the T-era, women with HER2 positive disease had a statistically significant lower risk of death compared to those with HER2 negative disease (HR, 0.49, 95% CI: 0.39, 0.62; p < 0.001). However, in the preT-era, HER2 overexpression was not significantly associated with survival (HR, 1.02, 95% CI: 0.66, 1.58, p = 0.92). Hormonal therapy was significantly associated with improved survival (HR, 0.62, 95% CI: 0.46, 0.84, p = 0.002) in the T-era compared to the preT-era (HR, 1.09, 95% CI: 0.73, 1.61, p = 0.68). Women with RPA class 3 had a higher risk of death compared to those with RPA class 1 (HR, 3.65; 95% CI, 2.52, 5.27; p < 0.001); RPA class 2 also had an elevated risk of death compared to class 1 (HR, 1.60; 95% CI, 1.12, 2.27; p = 0.009). Other significant prognostic factors included craniotomy use (p < 0.001), ER positive status (p = 0.001), chemotherapy use (p < 0.001), number of brain lesions (4+ lesions vs. 1, p = 0.017; 2–3 lesions vs. 1, p = 0.805) and SRS boost (p = 0.003).

**Table 2:** Multivariate analysis of overall survival including patients from both Pre-Trastuzumab and Trastuzumab eras

***Characteristics***	**HR(95% CI)**	***p-*value**
**RPA**		
1	1 [Reference]	
2	1.59 (1.12, 2.27)	0.009
3	3.64 (2.52, 5.27)	<0.001
**ER status**		
Negative	1 [Reference]	
Positive	0.67 (0.54, 0.84)	0.001
**Number of brain lesions**		
1	1 [Reference]	
2-3	1.04 (0.77, 1.41)	0.80
Multiple	1.36 (1.06, 1.75)	0.017
**Craniotomy**		
No	1 [Reference]	
Yes	0.43 (0.31, 0.58)	<0.001
**Chemotherapy**		
No	1 [Reference]	
Yes	0.59 (0.49, 0.73)	<0.001
**SRS boost**		
No	1 [Reference]	
Yes	0.40 (0.22, 0.73)	0.003
**HER2** (positive vs. negative)		
preT-era	1.02 (0.66, 1.58)	0.92
T-era	0.49 (0.39, 0.62)	<0.001
**Hormonal therapy** (yes vs. no)		
preT-era	1.09 (0.73, 1.61)	0.68
T-era	0.62 (0.46, 0.84)	0.002

*Abbreviations*: *RPA* = recursive partitioning analysis, *ER* = estrogen receptor, *SRS* = stereotactic radiosurgery, *HER2* = human epidermal growth factor receptor 2.

## Discussion

This population-based study evaluated outcomes and prognostic factors in patients with metastatic breast cancer treated with whole brain RT in a contemporary era compared to an era prior to the availability of trastuzumab.

Several retrospective studies have investigated on survival after the diagnosis of brain metastases in HER2 positive patients, and only one has included HER2 positive cases from a pre-trastuzumab era [12-16]. Eichler et al. identified 83 patients with breast cancer and new brain metastases diagnosed between 2001 and 2005, and found that the median survival from the time of brain metastases was 8.3 months. On univariate analysis, HER2 positive patients were found to have prolonged survival after brain metastases compared with HER2 negative patients (17.1 months vs 5.2 months) [15]. Bendell et al. identified 122 women with breast cancer treated with trastuzumab and reported that 42 patients (34%) developed cerebral disease with a median survival of 13 months from the time of brain metastases diagnosis [13]. Stemmler et al. reported on 42 patients with HER2 positive disease with a MS of 13 months after treatment with trastuzumab at the time of brain metastases [14]. In the analysis by Bartsch et al., 80 patients with brain metastasis from HER2-positive breast cancer were identified. In addition, a control group of 37 patients treated before 2003, when continuation of trastuzumab after diagnosis of brain metastasis was not yet advised, were also included [16]. They reported that the MS in patients receiving trastuzumab after diagnosis of BM was 13 months, compared to 3 months in patients treated with radiotherapy alone [16].

In the current series of 441 patients treated with whole brain RT in the T-era, a one-year OS of 26% compared to 12% for 128 patients treated in the preT-era was reported. Among HER2 negative patients, the 1-year OS (20%) and median survival was similar in the two eras (preT-era: 3.7 months; T-era: 3.2 months). This is comparable to the median OS of approximately 4 months reported in historical analyses of breast cancer patients with brain metastases [12,16-18]. However, for the HER2 positive patients, the absolute 1-year OS was significantly increased by 35% in the T-era from 5% to 40% and the median survival was increased by 3 months in the T-era from 3.4 months to 6.8 months.

The reasons behind the survival advantage for patients with HER2 positive disease may be explained by better systemic control outside the brain with trastuzumab or other systemic agents in the 2000s compared to the early 1990s [2]. It is also possible that trastuzumab itself contributes to local brain disease control by penetrating the brain at areas where the blood–brain barrier is disrupted following RT or that trastuzumab and RT have a synergistic effect on brain metastases.

On multivariable analysis, there were significant interactions between the era and both the use of hormonal therapy and HER2 expression. Survival was improved in the T-era for patients with HER2 overexpression and who received hormonal therapy. As the treatment era variable appears to interact with both HER2 overexpression and hormonal therapy, the interpretation of era effect on survival outcomes requires one to consider both HER2 status and hormonal therapy use. The era variable could not be reported as was done for terms that did not interact with other variables.

Moreover, the fact that 14% of the non-TMA cases were metastatic at initial diagnosis versus only 4% of the TMA cases implies that there may have been a referral bias to the frozen tissue archive used to make the TMA. However, if anything, the paucity of metastatic cases at presentation in the TMA cohort would tend to make the study outcomes better in the pre-T era, which was not observed. The absolute difference in 1-year OS following diagnosis of brain metastases was only 2% when comparing the TMA and non-TMA cases diagnosed in the preT-era. Therefore, possible TMA referral bias is unlikely to explain the difference in overall survival in the HER2 positive cases between the two eras.

There are several potential limitations in the current study. First, some of the data such as performance status were assigned retrospectively based on the description in the chart, decreasing the discrimination of the performance status categories. Data on toxicity could not be abstracted due to the limitations of a retrospective design. In addition, there is the possibility of lead time bias, in which greater use and access to cross-sectional, and particularly MRI imaging in the 2000s compared to 1990s may have led to an earlier diagnosis of brain metastases and hence contributed to the longer observed survival between eras.

The RPA prognostic index developed by Gaspar et al. was used in this study but did not account for the primary site as a prognostic parameter and did not include the number of brain lesions [11]. The significance of diagnosis-specific prognostic factors has been demonstrated in the Graded Prognostic Assessment (GPA) developed by Sperduto et al. [19-21]. Among patients with breast cancer, the only significant prognostic factor reported was the KPS [19-21]. The current study did not use the GPA index, since the use of the full range of KPS classifications was often not documented explicitly in the chart, as the case would be for most retrospective studies.

## Conclusion

This contemporary study demonstrates that subsequent to the availability of trastuzumab, the 1-year OS after brain RT was improved by 35% for HER2 positive patients compared to counterparts diagnosed and treated prior to the availability of trastuzumab. As survival was increased in the trastuzumab era only among patients with HER2 positive disease, this suggests that it is the use of trastuzumab or other factors, such as a possible beneficial interaction between RT and trastuzumab that have improved survival, rather than a characteristic of the biology, or natural history, of HER2 positive disease itself.

## Competing interests

The authors declare that they have no competing interests.

## Authors’ contributions

IK participated in the design of the study, performed the data collection and statistical analysis and drafted the manuscript. SH participated in the data collection and provided writing assistance of the manuscript. AN participated in the design of the study and provided writing assistance of the manuscript. RW performed the statistical analysis. CS participated in the data collection and analysis. HK participated in the design of the study and provided writing assistance. ST participated in the design of the study and data collection, performed the statistical analysis and provided writing assistance of the manuscript. All authors read and approved the final manuscript.

## References

[B1] Leyland-Jones B Human epidermal growth factor receptor 2–positive breast cancer and central nervous system metastases J Clin Oncol 2009 27 5278 5286 10.1200/JCO.2008.19.8481 19770385

[B2] Chia SK Speers CH D’yachkova Y Kang A Malfair-Taylor S Barnett J Coldman A Gelmon KA O’reilly SE Olivotto IA The impact of new chemotherapeutic and hormone agents on survival in a population-based cohort of women with metastatic breast cancer Cancer 2007 110 973 979 10.1002/cncr.22867 17647245

[B3] Tsao MN Lloyd NS Wong RK Rakovitch E Chow E Laperriere N Supportive Care Guidelines Group of Cancer Care Ontario’s Program in Evidence-based Care Radiotherapeutic management of brain metastases: a systematic review and meta-analysis Cancer Treat Rev 2005 31 256 273 10.1016/j.ctrv.2005.04.007 15951117

[B4] Tsao MN Lloyd N Wong R Chow E Rakovitch E Laperriere N Whole brain radiotherapy for the treatment of multiple brain metastases Cochrane Database Syst Rev 2012 4 CD003869 22513917 10.1002/14651858.CD003869.pub3 PMC6457607

[B5] Spector NL Blackwell KL Understanding the mechanisms behind Trastuzumab therapy for human epidermal growth factor receptor 2–positive breast cancer J Clin Oncol 2009 27 5838 5847 10.1200/JCO.2009.22.1507 19884552

[B6] Romond EH Perez EA Bryant J Suman VJ Geyer CE Davidson NE Tan-Chiu E Martino S Paik S Kaufman PA Swain SM Pisansky TM Fehrenbacher L Kutteh LA Vogel VG Visscher DW Yothers G Jenkins RB Brown AM Dakhil SR Mamounas EP Lingle WL Klein PM Ingle JN Wolmark N Trastuzumab plus adjuvant chemotherapy for operable HER2-positive breast cancer N Engl J Med 2005 353 1673 1684 10.1056/NEJMoa052122 16236738

[B7] Slamon D Leyland-Jones B Shak S Addition of herceptin [Superscript TM] (humazined anti-HER2 antibody) to first line chemotherapy for Her2 overexpressing metastatic breast cancer (HER2+/MBC) markedly increases anticancer activity: a randomized, multinational controlled phase III trial Proc Am Soc Clin Oncol 1998 17 98a

[B8] Lin NU Winer EP Brain metastases: the HER2 paradigm Clin Cancer Res 2007 13 1648 1655 10.1158/1078-0432.CCR-06-2478 17363517

[B9] Patchell RA Tibbs PA Walsh JW Dempsey RJ Maruyama Y Kryscio RJ Markesbery WR Macdonald JS Young B A randomized trial of surgery in the treatment of single metastases to the brain N Engl J Med 1990 322 494 500 10.1056/NEJM199002223220802 2405271

[B10] Chia S Norris B Speers C Cheang M Gilks B Gown AM Huntsman D Olivotto IA Nielsen TO Gelmon K Human epidermal growth factor receptor 2 overexpression as a prognostic factor in a large tissue microarray series of node-negative breast cancers J Clin Oncol 2008 26 5697 5704 10.1200/JCO.2007.15.8659 19001334

[B11] Gaspar L Scott C Rotman M Asbell S Phillips T Wasserman T McKenna WG Byhardt R Recursive partitioning analysis (RPA) of prognostic factors in three radiation therapy oncology group (RTOG) brain metastases trials Int J Radiat Oncol Biol Phys 1997 37 745 751 10.1016/S0360-3016(96)00619-0 9128946

[B12] Tham YL Sexton K Kramer R Hilsenbeck S Elledge R Primary breast cancer phenotypes associated with propensity for central nervous system metastases Cancer 2006 107 696 704 10.1002/cncr.22041 16826579

[B13] Bendell JC Domchek SM Burstein HJ Harris L Younger J Kuter I Bunnell C Rue M Gelman R Winer E Central nervous system metastases in women who receive trastuzumab-based therapy for metastatic breast carcinoma Cancer 2003 97 2972 2977 10.1002/cncr.11436 12784331

[B14] Stemmler H Heinemann V Central nervous system metastases in HER-2-overexpressing metastatic breast cancer: a treatment challenge Oncologist 2008 13 739 750 10.1634/theoncologist.2008-0052 18614587

[B15] Eichler AF Kuter I Ryan P Schapira L Younger J Henson JW Survival in patients with brain metastases from breast cancer The importance of HER-2 status Cancer 2008 112 2359 2367 10.1002/cncr.23468 18361426

[B16] Bartsch R Berghoff A Pluschnig U Bago-Horvath Z Dubsky P Rottenfusser A DeVries C Rudas M Fitzal F Dieckmann K Mader RM Gnant M Zielinski CC Steger GG Impact of anti-HER2 therapy on overall survival in HER2-overexpressing breast cancer patients with brain metastases Br J Cancer 2012 106 25 31 10.1038/bjc.2011.531 22127284 PMC3251869

[B17] DiStefano A Yong Yap Y Hortobagyi GN Blumenschein GR The natural history of breast cancer patients with brain metastases Cancer 1979 44 1913 1918 10.1002/1097-0142(197911)44:5<1913::AID-CNCR2820440554>3.0.CO;2-D 498057

[B18] Boogerd W Vos VW Hart AA Baris G Brain metastases in breast cancer; natural history, prognostic factors and outcome J Neurooncol 1993 15 165 174 10.1007/BF01053937 8509821

[B19] Sperduto PW Berkey B Gaspar LE Mehta M Curran W A new prognostic index and comparison to three other indices for patients with brain metastases: an analysis of 1,960 patients in the RTOG database Int J Radiat Oncol Biol Phys 2008 70 510 514 10.1016/j.ijrobp.2007.06.074 17931798

[B20] Sperduto PW Chao ST Sneed PK Luo X Suh J Roberge D Bhatt A Jensen AW Brown PD Shih H Kirkpatrick J Schwer A Gaspar LE Fiveash JB Chiang V Knisely J Sperduto CM Mehta M Diagnosis-specific prognostic factors, indexes, and treatment outcomes for patients with newly diagnosed brain metastases: a multi-institutional analysis of 4,259 patients Int J Radiat Oncol Biol Phys 2010 77 655 661 10.1016/j.ijrobp.2009.08.025 19942357

[B21] Sperduto PW Kased N Roberge D Xu Z Shanley R Luo X Sneed PK Chao ST Weil RJ Suh J Bhatt A Jensen AW Brown PD Shih HA Kirkpatrick J Gaspar LE Fiveash JB Chiang V Knisely JP Sperduto CM Lin N Mehta M Summary report on the graded prognostic assessment: an accurate and facile diagnosis-specific tool to estimate survival for patients with brain metastases J Clin Oncol 2012 30 419 425 10.1200/JCO.2011.38.0527 22203767 PMC3269967

